# Quality of Life of Taiwanese Adults with Autism Spectrum Disorder

**DOI:** 10.1371/journal.pone.0109567

**Published:** 2014-10-09

**Authors:** Ling-Yi Lin

**Affiliations:** Department of Occupational Therapy, College of Medicine, National Cheng Kung University, Tainan, Taiwan; Hamamatsu University School of Medicine, Japan

## Abstract

**Background:**

To date, few recent studies have investigated the quality of life of adults with autism spectrum disorder (ASD). It remains unclear how individuals with ASD view their own quality of life.

**Objective:**

The primary purpose of this study was to compare the quality of life scores among adults with ASD with those of a non-ASD control group and the Taiwanese health population reference group.

**Methods:**

The study comprised 41 adults with ASD (M age = 26.9, SD = 5.0), and without intellectual disabilities (IQ>70). A comparison sample of 41 adults without ASD was selected by matching the age and sex of the participants with ASD. A validated measure, the Taiwanese version of the World Health Organization Quality of Life-BREF (WHOQOL-BREF), was used. Independent t-tests were performed to examine the differences in the quality of life between groups.

**Results:**

The highest quality of life was scored in the environment domain, followed by the physical health and psychological health domains. The lowest quality of life score was found in the social relationship domain. Adults with ASD scored significantly lower in all domains than did the non-ASD control group. Additionally, adults with ASD scored significantly lower in the physical health, psychological health, and social relationship domains than did the Taiwanese health population reference group. Comorbid psychiatric disorders, self-rated health status, and perceived happiness were correlated with quality of life among adults with ASD.

**Conclusion:**

The preliminary findings suggest that adults with ASD need more supportive social contexts and interventions to promote their quality of life. Based on our findings, social relationship must be considered in designing and applying treatment programs for adults with ASD.

## Introduction

Autism spectrum disorder (ASD) is characterized by deficits in social-emotional reciprocity, nonverbal communicative behaviors used for social interaction, and developing and maintaining relationships across various contexts, and the presence of restricted repetitive patterns of behavior, interests, or activities [1]. The Centers for Disease Control and Prevention in the United States estimated that an average of 1 in 68 children has ASD [2]. The prevalence of ASD is reportedly increasing in Western countries as well as in Taiwan [3]. In 2007, the annual rate of increase in ASD was 16.52%, which was the highest among all disabilities [4]. The number of identified persons with ASD in Taiwan was 13,366 in 2013 [5]. Almost 25% of those with ASD were 18 years old and older. Within 10 years, approximately 7,000 more people in Taiwan with ASD will become 18 years old and older creating increasing challenge in adult placement. Many experts and researchers across nations have started to address the issues and problems faced by adults with ASD [6]–[8].

Increasing attention has been paid to studying the outcomes of individuals with ASD during adolescence and adulthood in Western countries [9]. Functional independence and employment are major factors related to outcomes in adulthood. Research in Western countries indicates that many adults with ASD need high levels of assistance and care [10]–[13]. Previous studies also reported that people with ASD had poor outcomes in adulthood and a low rate of employment is common for adults with ASD across different countries [10], [13]–[16].

Quality of life refers to an individual's general wellbeing, including emotional, social, and physical aspects of the individual's life [17]. Previous studies have indicated that quality of life is associated with various health conditions (such as pain, mental health problems) among individuals with disabilities [18], [19]. Over recent years, quality of life has become a critical measure of treatment outcome for people with mental and physical health concerns. Researchers systematically reviewed literature and suggested using quality of life as indicators to evaluate outcomes for adults with ASD [20], [21]. They suggest that practitioners can effectively establish person-centered planning in service delivery when a person with ASD provides his or her subjective quality of life. This may lead to more effective intervention.

While some studies [22]–[31] on quality of life in adults with ASD have been published in recent years, only a handful of recent studies have investigated the quality of life of adults with ASD by using a cross-culturally standardized self-reported instrument (e.g., the WHOQOL-BREF questionnaire) [25]–[27]. Previous studies using the WHOQOL-BREF questionnaire have yielded inconsistent results. Jennes-Coussens and colleagues [25] evaluated 13 young adults with Asperger syndrome and found lower quality of life in the domains of physical health and social relationship when compared with healthy controls. They reported that no differences in the psychological and environmental domains were observed, whereas Kamp-Becker and colleagues [27] indicated that individuals with ASD have impairments in the psychological health domain and the overall quality of life score. In Japan, Kamio and colleagues [26] only examined the psychological and social aspects of quality of life in the participants with ASD, indicating that individuals with ASD reported significantly lower scores of the psychological and social domains than those of the healthy Japanese population. However, no studies have addressed the quality of life of adults with ASD in Taiwan or China.

This study addressed a major concern in the existing published research: the quality of life of adults with ASD. The aims of the study were to examine the quality of life of adults with ASD in Taiwan, and to compare with the quality of life of a health control group and the Taiwanese health population reference group. Thus, in the current study, we addressed the following questions: (1) How do adults with ASD rate their own quality of life? (2) How do adults with ASD differ from adults without ASD and the Taiwanese health population reference group with respect to their views on quality of life? (3) How is quality of life related to age, gender, education level, employment status, smoking habits, drinking habits, comorbid psychiatric disorders, self-rated health status, and perceived happiness?

## Methods

### Design

A cross-sectional survey was undertaken during the period August 2013 to September 2013.

### Procedure

Ethical clearance for the study was received from the National Cheng Kung University Hospital internal review board (A-BR-101-074). Adults with ASD were recruited from the hospital clinic and local autism groups by using fliers. Adults without ASD were recruited using study flyers that were posted on college campuses and in libraries, local supermarkets, and other public areas. The investigators fully explained the procedures to all of the participants, from whom written informed consent was obtained before enrolling in the study.

### Participants

#### Adults with ASD

Forty-one individuals with ASD (mean age 26.9, range 20–37 years) were recruited for this study. There were 30 males and 11 females. All the participants had been diagnosed with autism spectrum disorders by a registered psychiatrist using the Diagnostic and Statistical Manual IV Text Revision (DSM-IV-TR) criteria [32]. Intellectual disabilities (IQ<70) were excluded. Thirty-five participants were diagnosed with Asperger syndrome and six individuals were diagnosed with pervasive developmental disorder not otherwise specified (PDD-NOS). In order to determine the reading and writing skills of the participants, the participants were asked to read a paragraph of the newspaper. Immediately after reading the paragraph, participants were asked to write five words that exist in the paragraph. All adults with ASD were able to read and write Mandarin words and phrases.

#### Adults without ASD

The control group comprised 122 adults without ASD from the community. They were recruited through advertisement in the local communities. The exclusion criteria were (a) any developmental disabilities; and (b) intellectual disabilities (IQ<70). A comparison sample was selected by matching the age and sex of the adults without ASD with those of the ASD group. Finally, we selected 41 adults without ASD. There were 30 males and 11 females, with a mean age of 26.9 years. All adults without ASD were able to read and write Mandarin words and phrases. Table 1 lists the sample characteristics. There were significant differences in educational level, employment status, comorbid psychiatric disorders, and drinking habits between ASD and non-ASD groups.

**Table 1:** Demographic Characteristics of Participants.

Characteristics	ASD M (*SD*) or n (%)	Non-ASD M (*SD*) or n (%)	Statistic
Gender			
Male	30 (73.2%)	30 (73.2%)	−
Female	11 (26.8%)	11 (26.8%)	
Age (yr), mean±SD	26.9 (5.0)	26.9 (5.0)	−
Educational level			
High school and below	10 (24.4%)	1 (2.4%)	?^2^ = 8.5**
College and above	31 (75.6%)	40 (97.6%)	
Employed	20 (48.8%)	29 (70.7%)	?^2^ = 4.1**
Marital status			
Single	39 (95.1%)	35 (85.4%)	?^2^ = 2.2
Married/Living together	2 (4.9%)	6 (14.6%)	
Comorbid psychiatric disorders	8 (19.5%)	0	?^2^ = 8.9**
Smoking	2 (4.9%)	4 (9.8%)	?^2^ = 0.7
Drinking	13 (31.7%)	23 (56.1%)	?^2^ = 4.9*

*p<.05;

**p<.01.

### Measures

#### Demographic information

The demographic characteristics included age, sex, diagnosis, education level, employment status, occupation, marital status, comorbid psychiatric disorders, and smoking and drinking habits.

#### WHOQOL-BREF Taiwan version

The Taiwanese version of the WHOQOL-BREF contains 28 items classified into the same four domains (physical health, psychological health, social relationship, and environmental) as the standard WHOQOL-BREF questionnaire [33]. Items were scored on a five-point scale to determine a raw item score. Raw item scores can be transformed the domain scores (range from 4 to 20). The transformed domain scores were used in the analyses. Higher transformed scores indicate better quality of life. The WHOQOL-BREF Taiwan version has been shown to be internally consistent and to have strong construct validity [34]. The internal consistency coefficients ranged from 0.78 to 0.88 for the four domains for the sample in the present study.

#### Self-rated health status and perceived happiness

The participants were asked to rate two 5-point Likert scale questions: “How do you rate your overall health?” and “Overall, how happy do you feel currently?” Self-rated health status was rated from 1 (extremely bad) to 5 (extremely good). Perceived happiness was rated from 1 (extremely unhappy) to 5 (extremely happy).

### Data analysis

We used SPSS 17.0 for Windows (SPSS Inc., Chicago, IL) to analyze the data and descriptive statistics to examine the demographic data and outcome measures for the study variables. Independent *t*-tests and χ^2^ tests were performed to examine the differences between group demographic data and outcome measures. A series of ANCOVAs was conducted to examine whether differences in demographic characteristics accounted for differences in four domains in the two groups. Fisher's exact tests were used when more than 20% of the values were less than the expected value of 5 in a contingency table. Bivariate correlations were computed to identify significant relationships among all variables. A *p*-value of.05 was considered significant.

## Results

Table 2 lists the WHOQOL-BREF results for the present sample. For adults with ASD, the mean total score was 49.5±11.6. The highest quality of life score was reached in the environment domain (13.5±2.8), followed by the physical health (13.1±3.4) and the psychological health (11.8±3.5) domains. The lowest quality of life score was in the social relationship domain (11.1±3.5). Within the ASD group, the scores of the environment and physical health domains were significantly higher than scores on the psychological health and social relationship domains.

**Table 2:** Quality of Life, Self-rated Health Status, and Perceived Happiness among Adults with and without ASD.

Variables	ASD M (*SD*) or n (%)	Non-ASD M (*SD*) or n (%)	Statistics
Overall quality of life	49.5 (*11.6*)	58.1 (*6.0*)	*t* = −4.2***
Physical health	13.1 (*3.4*)	15.3 (*1.6*)	*t* = −3.7**
Psychological health	11.8 (*3.5*)	14.0 (*2.0*)	*t* = −3.7**
Social relationship	11.1 (*3.5*)	14.1 (*1.5*)	*t* = −5.0***
Environment	13.5 (*2.8*)	14.7 (*1.9*)	*T* = −2.2*
Self-rated health status			
Extremely bad	5 (12.2%)	0	?^2^ = 11.9*
Bad	6 (14.6%)	3 (7.3%)	
Not bad/Not good	16 (39.0%)	13 (31.7%)	
Good	7 (17.1%)	19 (46.3%)	
Extremely good	7 (17.1%)	6 (14.6%)	
Self-rated perceived happiness			
Extremely unhappy	3 (7.3%)	0	?^2^ = 23.3***
Unhappy	12 (29.3%)	1 (2.4%)	
Fair	17 (41.5%)	14 (34.1%)	
Happy	5 (12.2%)	22 (53.7%)	
Extremely happy	4 (9.8%)	4 (9.8%)	

*p<.05;

**p<.01;

***p<.001.

For non-ASD control group, the mean total score was 58.1±6.0. The highest quality of life score was reached in the physical health domain (15.3±1.6), followed by the environment (14.7±1.9) and social relationship (14.1±1.5) domains. The lowest quality of life score was in the psychological health domain (14.0±2.0). There were no significant differences in the scores of the four domains within the non-ASD control group.

**Figure 1:**
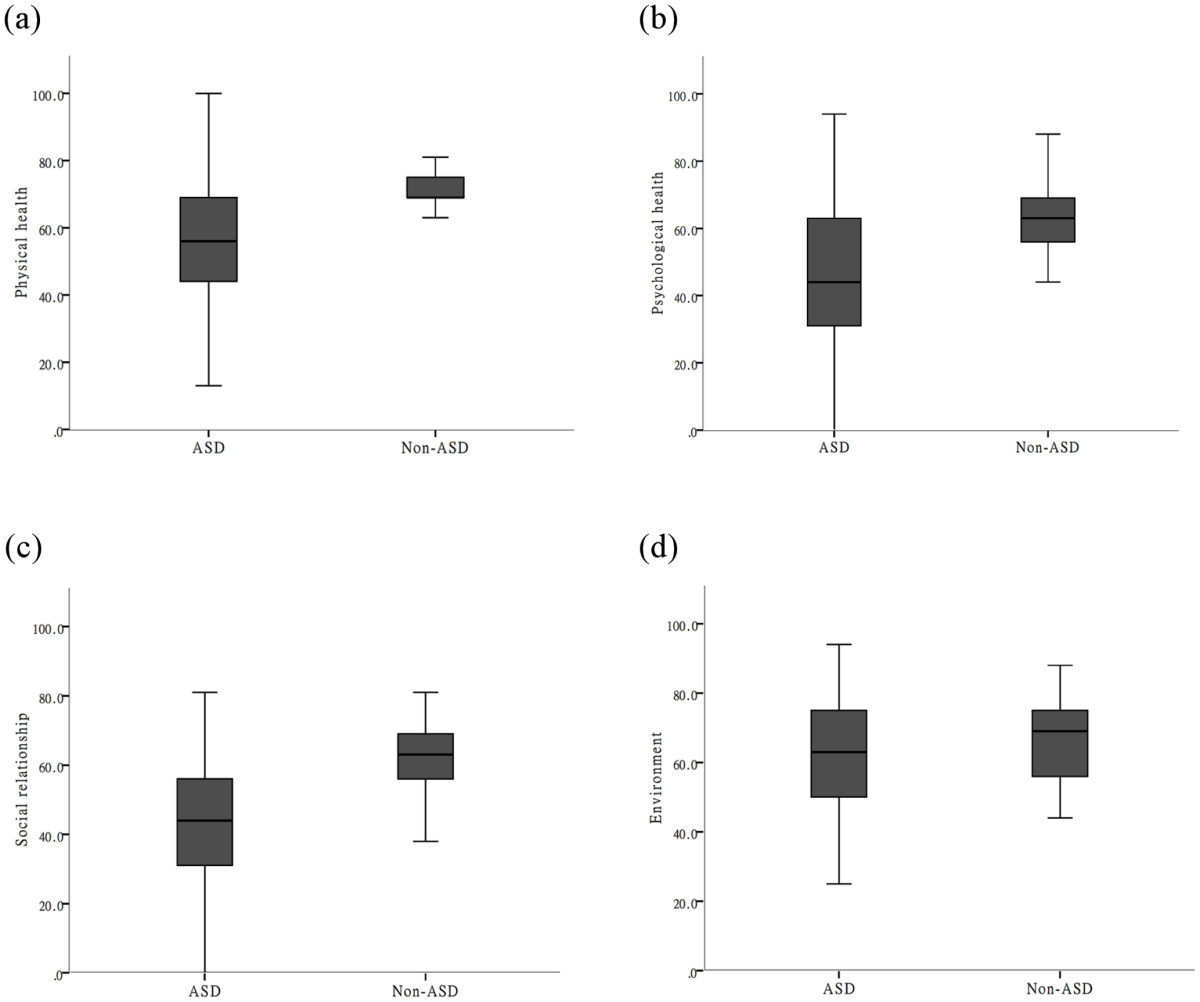
The Variability of Four Domains among People in Each Group.

When comparing the WHOQOL-BREF scores of adults with ASD to a non-ASD control group, the scores of the participants with ASD were significantly lower in all domains and the overall score. The results indicated that adults with ASD rated their quality of life lower than did adults without ASD. Figure 1 shows the variability among people in each group.

There were significantly differences in the educational level, employment status, comorbid psychiatric disorders, and drinking habits between the two groups. The educational level, employment status, comorbid psychiatric disorders, and drinking habits were examined independently as covariates. When the educational level, comorbid psychiatric disorders, and drinking habits were entered separately as covariates, the observed differences in the environmental domain became nonsignificant. Once the employment status was entered into the model as a covariate, the observed differences in the environmental domain remained significant. The observed differences in domains of physical health, psychological health, and social relationship remained significant when these variables were entered as covariates.

The Taiwanese health population reference group comprised 9107 health individuals, with a mean age of 36.1 years [33]. The mean values of the physical health, psychological, social relationship, and environmental domains of the WHOQOL-BREF among the Taiwanese health population reference group were 15.31±1.93, 13.80±2.19, 14.22±2.05, and 13.33±2.05, respectively. Compared to the Taiwanese health population reference group, our ASD sample scored lower in all WHOQOL-BREF domains. The mean scores of the physical and psychological health domains were one standard deviation below the normative population mean. The average score of the social relationship domain was 1.5 standard deviations below the normative population mean. The mean score of the environment domain was close to the normative population mean.

Table 2 also shows self-rated health status and perceived happiness of the participants. Eleven adults with ASD rated their health status as bad and extremely bad. Fourteen individuals rated their health status as good and extremely good. The rest of the participants reported the health status as not good and not bad. Fifteen adults with ASD reported perceived unhappiness and extreme unhappiness. Only nine individuals reported perceived happiness as happy and extremely happy. Adults with ASD were less likely to rate their health status as good and extremely good (34.1% vs. 61.0%) and perceived happiness as happy and extremely happy (22.0% vs. 63.4%) than adults without ASD.

In addition, it is found that age, gender, level of education, and employment status were not related to quality of life for adults with ASD and without ASD. Comorbid psychiatric disorders (r = −.44, *p*<.01), self-rated health status (r = .82, *p*<.001), and perceived happiness (r = .78, *p*<.001) were positively related to quality of life among adults with ASD. For non-ASD group, drinking (r = −.47, *p*<.01), subjective health status (r = .41, *p*<.05), and perceived happiness (r = .75, *p*<.001) were related to quality of life. Overall, self-rated health status and perceived happiness were correlated with quality of life for both adults with and without ASD.

## Discussion

This study examined the quality of life of adults with ASD, as compared with the quality of life of a non-ASD control group and the Taiwanese health population reference group. We determined three primary findings. First, the lowest quality of life score for ASD participants was in the social relationship domain. Second, participants with ASD scored significantly lower in all domains than did the non-ASD control group. Third, comorbid psychiatric disorders, self-rated health status, and perceived happiness were correlated with quality of life among adults with ASD. No significant relationships were found between other demographic characteristics and quality of life.

Consistent with the studies of Jennes-Coussens et al. [25] and Kamp-Becker et al. [27], we found that the lowest quality of life score was observed in the social relationship domain. As expected, adults with ASD rated the lowest score in the social relationship domain, which reflects their core symptoms of ASD. The second last scores were in the psychological health domain. Kamio et al. [26] and Kamp-Becker et al. [27] indicated that adults with ASD having comorbid psychiatric disorders would report lower scores in the psychological health domain. In this study, we found that eight individuals with ASD had comorbid psychiatric disorders such as depression, anxiety, and obsessive compulsive disorder.

Consistent with a previous study [27], we found that adults with ASD had significantly lower levels of overall quality of life than those of a non-ASD control group. Specifically, our results confirmed the findings of three previous studies [25]–[27], which have indicated that adults with ASD scored lower in the social domain than did those without ASD. These individuals with ASD were aware of their social disability and perceived their social deficits to impact negatively on their quality of life. This suggests that adults with ASD need support to facilitate and maintain social functioning. Designing and applying social relationship treatment programs should be considered for adults with ASD.

Adults with ASD in the present study rated scores lower in physical health than the non-ASD controls, which was consistent with previous studies [25], [27]. Although impairments in physical health are not key symptoms of ASD, previous studies have indicated that factors such as daily living skills and sensory modulation problems may influence the physical health scores among adults with ASD [25], [27]. This suggests that potential factors associated with quality of life should be the focus of future investigations.

Previous studies have not reported significant differences in the environmental domain between ASD and non-ASD groups [25], [27]. By contrast, adults with ASD in the current study rated lower scores in environmental domain than non-ASD participants. The environmental domain comprises the perception of security, financial burden, home environment, availability of medical and social care, availability of new information, acquisition of skills, participation in recreation or leisure, transportation, and other environmental factors [33]. Over half of adults with ASD in the current study were unemployed. Adults with ASD were employed typically lived on small salaries [10], [13], [15]. The rate of college and university attendance was substantially low for adults with ASD [15], [16]. Additionally, adults with ASD may have difficulties in engaging in recreational and leisure activities because of a lack of adequate autism-focused adult services [16]. This may explain the low scores in the environmental domain rated by adults with ASD.

Furthermore, the same variables related to quality of life were found for both adults with and without ASD. They reported that self-rated health status and perceived happiness were correlated with their quality of life. Our findings are in line with the reports of Burgess and Gutstein [20] and Renty and Roeyers [29] who showed that subjective perspectives of individuals with ASD are crucial indicators of quality of life. Future research is needed.

The major limitations of this study were the small sample size and purposive sampling. The investigator acknowledges that the small sample size may have generated biased the results. In addition, inferential statistics such as the regression analysis were not conducted due to the small sample size. Future studies should use larger samples and instruments of potential factors.

## Conclusions

In summary, adults with ASD rated their quality of life significantly lower than adults without ASD did. The preliminary findings of this study are relevant for interventions in adults with ASD. Adults with ASD in the present study demonstrated a clear need to improve their social ability to function in and adapt to society. The low scores in the social relationship domain indicate that therapeutic interventions are required for adults with ASD. Social relationship should be a focus in designing and applying treatment programs. Additionally, practitioners have to consider the presence of comorbid psychiatric disorders among some adults with ASD to address their psychological well-being. The potential factors associated with quality of life highlight new questions that are worthy of future research. Replication of these findings with larger samples is needed.
